# MMP-9 plasma level as biomarker of cochlear implantation outcome in cohort study of deaf children

**DOI:** 10.1007/s00405-023-07924-y

**Published:** 2023-04-01

**Authors:** Monika Matusiak, Dominika Oziębło, Monika Ołdak, Emilia Rejmak, Leszek Kaczmarek, Dominik Dobek, Henryk Skarżyński

**Affiliations:** 1 Oto-Rhino-Laryngosurgery Clinic, Institute of Physiology and Pathology of Hearing, M Mochnackiego 10, 02-042 Warsaw, Poland; 2 World Hearing Centre, Mokra 17, 05-830 Nadarzyn, Poland; 3 Department of Genetics, Institute of Physiology and Pathology of Hearing, M Mochnackiego 10, 02-042 Warsaw, Poland; 4 BRAINCITY, Nencki Institute of Experimental Biology, L Pasteura 3, 02-093 Warsaw, Poland; 5 Transition Technologies Science, Pawia 55, 01-030 Warsaw, Poland

**Keywords:** Neuroplasticity, MMP-9, Cochlear implant, Outcome biomarker

## Abstract

**Purpose:**

If before cochlear implantation it was possible to assay biomarkers of neuroplasticity, we might be able to identify those children with congenital deafness who, later on, were at risk of poor speech and language rehabilitation outcomes.

**Methods:**

A group of 40 children aged up to 2 years with DFNB1-related congenital deafness was observed in this prospective cohort study over three follow-up intervals (0, 8, and 18 months) after cochlear implant (CI) activation. Children were assessed for auditory development using the LittlEARS Questionnaire (LEAQ) score, and at the same time, measurements were made of matrix metalloproteinase-9 (MMP-9) plasma levels.

**Results:**

There were significant negative correlations between plasma levels of MMP-9 at 8-month follow-up and LEAQ score at cochlear implantation (*p* = 0.04) and LEAQ score at 18-month follow-up (*p* = 0.02) and between MMP-9 plasma levels at 18-month follow-up and LEAQ score at cochlear implantation (*p* = 0.04). As already reported, we confirmed a significant negative correlation between MMP-9 plasma level at cochlear implantation and LEAQ score at 18-month follow-up (*p* = 0.005). Based on this latter correlation, two clusters of good and poor CI performers could be isolated.

**Conclusions:**

The study shows that children born deaf who have an MMP-9 plasma level of less than 150 ng/ml at cochlear implantation have a good chance of attaining a high LEAQ score after 18 months of speech and language rehabilitation. This indicates that MMP-9 plasma level at cochlear implantation is a good prognostic marker for CI outcome.

## Introduction

### Variability of cochlear implantation outcome

For over 30 years, congenital deafness has been routinely treated with cochlear implantation, and the majority of implanted children become regular cochlear implant (CI) users [1]. However, despite the fact that current, technologically advanced CIs employ optimal electrical stimulation and speech processing strategies, there is still enormous variation in treatment outcomes [2, 3]. To a certain extent, this can be accounted for by already known factors such as coexisting disabilities, age at implantation, deafness etiology, and others [2, 4–7]. The most common genetic factor for cochlear malfunction is the presence of a congenital pathogenic variant of the *DFNB1* locus on chromosome 13 [8]. Given the complex interplay between the implant and the auditory cortex, and the known effect of brain plasticity, there must be some additional molecular factors at work which control how well the brain adapts to the prosthesis and affect the functional outcome of cochlear implantation [3, 9].

### Neuroplasticity after auditory deprivation

Delivery of electrical currents to spiral ganglion nerve fibers enables maturation of the auditory cortex by promoting synapse formation and remodeling [3, 10]. Synaptic plasticity reflects the molecular underpinnings of brain connectivity and promotes the learning and memory processes that increase the computational power of the brain [3, 11–14]*.* Since plasticity varies greatly between individuals, the hope has been that a certain set of biomarkers might be identified that reflect the brain’s capacity for plastic remodeling. In this way, the biomarker could be used by clinicians as a tool to identify a child who was at risk of poor speech and language rehabilitation by employing more patient-specific management [3, 10, 15]. In our previous studies, a first attempt was made to identify such a biomarker [16].

### Molecular background to neuroplasticity

Accumulating evidence suggests that human brain plasticity during critical, developmental periods is regulated by cascade mechanisms involving multiple molecular agents in synaptic remodeling [11, 14, 17–19]. One of the best studied molecules having a well-established role in long-term potentiation (LTP)—a key phenomenon that underlies molecular mechanisms of synaptic plasticity—is matrix metalloproteinase-9 (MMP-9) [11, 12, 14–23]. Results of human studies suggest that MMP-9 is involved in the neuroplasticity associated with various neuropsychiatric conditions and functional responses to cochlear implantation following the period of auditory deprivation caused by congenital deafness [12, 14, 15, 24–26]. Relevant here, our previous studies have indicated a predictive role for rs3918242 *MMP9* functional polymorphism in treatment outcomes, and have reported a significant negative correlation between MMP-9 plasma level measured at cochlear implantation and later auditory development outcome following 18 months of CI use [15, 16]*.* However, despite the fact that published reports do not provide detailed data on natural changes of MMP-9 plasma levels in the early years of life, the existing evidence does indicate that a decrease in MMP-9 levels correlates positively with higher degrees of neuroplasticity [11, 12, 14, 27–29].

It is of great interest to gain a more detailed insight into how MMP-9 contributes to auditory development following congenital deafness treatment with cochlear implantation. To the best of our knowledge, there are no reports of possible molecular factors contributing to the regulation of neuroplasticity in this clinical condition. Here, we report the findings of a prospective cohort study in which longitudinal observations of auditory development were made with the LittlEARS Questionnaire (LEAQ) while simultaneously measuring serum levels of MMP-9. The cohort consisted of 40 children with DFNB1-related deafness who underwent cochlear implantation before their 2nd birthday, and measurements were performed then and at two subsequent intervals.

### Aim of the study

The study aimed to confirm the hypothesis that there is a certain level of MMP-9 in plasma, measured at cochlear implantation, which can serve as a biomarker of good speech and language rehabilitation after 18 months of CI use. A second hypothesis was that plasma levels of MMP-9 in the same group of children, again measured at cochlear implantation and at follow-up intervals, were significantly correlated with simultaneously collected LEAQ scores.

## Materials and methods

### Study design and participants

Between December 2016 and December 2019, we enrolled children with congenital deafness to a study group at the Institute of Physiology and Pathology of Hearing, Warsaw, Poland. They were infants and toddlers who had undergone the routine procedure of cochlear implantation with full insertion of the array, performed by the same surgeon and with the same type of device, and had their speech processor activated before the age of 2. All children had pathogenic variants in the DFNB1 locus and were diagnosed with congenital bilateral profound sensorineural hearing loss which was confirmed by auditory brainstem responses (ABRs)*.* We excluded children with any chronic concomitant disease, including developmental delay, or with a history of severe prematurity, asphyxia, or viral infection during pregnancy. After CI activation, parents or caregivers were instructed in auditory/verbal therapy. All children followed the same observation program, which consisted of measurements of plasma MMP-9 and C-reactive protein (CRP) performed at cochlear implantation and at 8 and 18 months after CI activation (MMP-9_0, MMP-9_8, MMP-9_18). Auditory development measures were assessed using the LittlEARS Auditory Questionnaire (LEAQ) before CI activation and at the 8th and 18th month after CI activation (LEAQ_0, LEAQ_8, LEAQ_18). From the original group of 45 children, 5 were excluded due to elevated CRP level or parents withdrawing them from the study. The final study group of 40 children was sorted according to age at CI activation. Demographic data were collected from all participants, who were all of Caucasian origin. Plasma samples and LEAQ scores were collected from all participants at all three intervals. The study was designed and conducted according to the Declaration of Helsinki and was reviewed and approved by the Bioethics Committee of the Institute of Physiology and Pathology of Hearing (no. IFPS:KB/13/2015). Parents or caregivers of all children gave written informed consent.

### Auditory development assessment

We used LEAQ in our study to assess early stages of auditory development [30]. LEAQ consists of 35 questions with a yes or no answer, with a final score made up of the total number of yes answers. LEAQ has been widely validated in many languages [31–36].

### Plasma sample collection

Blood samples were collected in heparin tubes and centrifuged at 1400*g* for 15 min. Plasma was then collected, aliquoted, and stored at − 80 °C for further analysis. Total protein content was measured by a BCA protein assay kit (Thermo-Scientific) following the manufacturer’s protocol.

### MMP-9 plasma levels

The levels of MMP-9 in the plasma samples were measured using commercially available specific enzyme-linked immunosorbent assay (ELISA) kit (R&D Systems Inc., Minneapolis, USA). Assays were performed according to the manufacturer’s instructions. A total of 30 μg/μl of protein from each plasma sample was diluted 70-fold with calibration diluent from the assays and analyzed in duplicate. Absorbances were measured at 450 nm using an automated microplate reader (Sunrise Microplate Absorbance Reader).

### Statistical analyses

#### Correlation analysis methodology

LEAQ scores and MMP-9 levels, measured at different time intervals from CI activation, were tested for correlation presence and strength using a Pearson test (if test assumptions were met) or a Spearman test (if not). Prior to correlation tests, a Shapiro–Wilk test of normality was made in order to check assumptions. All variables for which the correlation was tested were normalized using the min–max scaling method. A correlation was considered statistically significant at a *p*-value ≤ 0.05. All computations were made using R version 3.6.3 (2020).

#### Clustering methodology

Partitioning the data into two clusters was done using the PAM (Partitioning Around Medoids) algorithm, which is similar to the K-means algorithm [37]. The main difference between these algorithms is that the PAM algorithm uses medoids (observations from the dataset are used to form cluster “centers”) instead of centroids. The advantage of this algorithm is its resistance to outliers. Clustering was prepared using two variables, i.e., LEAQ_18 scores and MMP-9_0. Prior to the clustering process, both variables were scaled. Paired comparisons were prepared for individual clusters as well as between them. All computations were made using R version 3.6.3 (2020).

#### Paired comparisons methodology

For all tested follow-up intervals, comparisons of mean LEAQ score and MMP-9 level were made between patients belonging to the two clusters using a Welch two-sample *t*-test (if test assumptions were met) or a Wilcoxon rank-sum test (if not). All calculations were performed with the R language (version 3.6.3). Results were considered statistically significant at a *p*-value ≤ 0.05.

## Results

### Sample demographics and auditory development

The study group’s demographics were described in detail in our first paper [16]. Briefly, 18 children were girls (45%), 22 were boys (55%), and the mean age at CI activation was 407.7 days (min = 208, max = 654, SD = 128). All subjects were implanted with a Med-El Synchrony CI and became regular CI users. The LEAQ scores for the study group of 40 children are shown in Table 1.

**Table 1:** Plasma levels of MMP-9 and LEAQ scores at all three follow-up intervals

MMP-9_0 [ng/ml]				MMP-9_8 [ng/ml]				MMP-9_18 [ng/ml]			
Mean	Min	Max	SD	Mean	Min	Max	SD	Mean	Min	Max	SD
223.54	31.1	456.7	107.1	137.3	10.3	490.4	123.8	121.4	0.6	512.3	103.7

LEAQ_0				LEAQ_8				LEAQ_18			
Mean	Min	Max	SD	Mean	Min	Max	SD	Mean	Min	Max	SD
5.5	0	28	6.67	27.17	9	35	5.75	32.57	24	35	3.19

*min* minimum value, *max* maximum value, *SD* standard deviation

### MMP-9 plasma levels

The plasma levels of MMP-9 for the study group are shown in Table 1. The mean value of MMP-9 plasma concentration was highest at cochlear implantation, with subsequent decreases in the following intervals.

### Analyses in the study group

To assess the possible effect of MMP-9 on auditory development, correlations between plasma levels of MMP-9 at all three follow-up intervals and LEAQ scores measured at the same time were tested. Four correlations reached statistical significance, and two of them show the predictive value of MMP-9 plasma levels at cochlear implantation and at 8-month follow-up for LEAQ_18. Table 2 shows that there were significant negative correlations between plasma levels of MMP-9_0 and LEAQ_18 (*p* = 0.005) and between MMP-9_8 and LEAQ_18 (*p* = 0.02).

**Table 2:** Correlations between LEAQ score at three follow-up intervals and plasma levels of MMP-9

Plasma level of:	LEAQ_0	LEAQ_8	LEAQ_18
MMP-9_0	*p* = 0.9 rho = − 0.0003	*p* = 0.07, rho = − 0.2	*p* = 0.005, rho = –0.4*
MMP-9_8	***p* = 0.04, rho = –0.3**	*p* = 0.2, rho = − 0.2	***p* = 0.02, rho = –0.3**
MMP-9_18	***p* = 0.04, rho = –0.3**	*p* = 0.2, rho = − 0.1	*p* = 0.2, rho = − 0.2

*ref. (Matusiak & Oziębło et al. 2021)

**Fig. 1:**
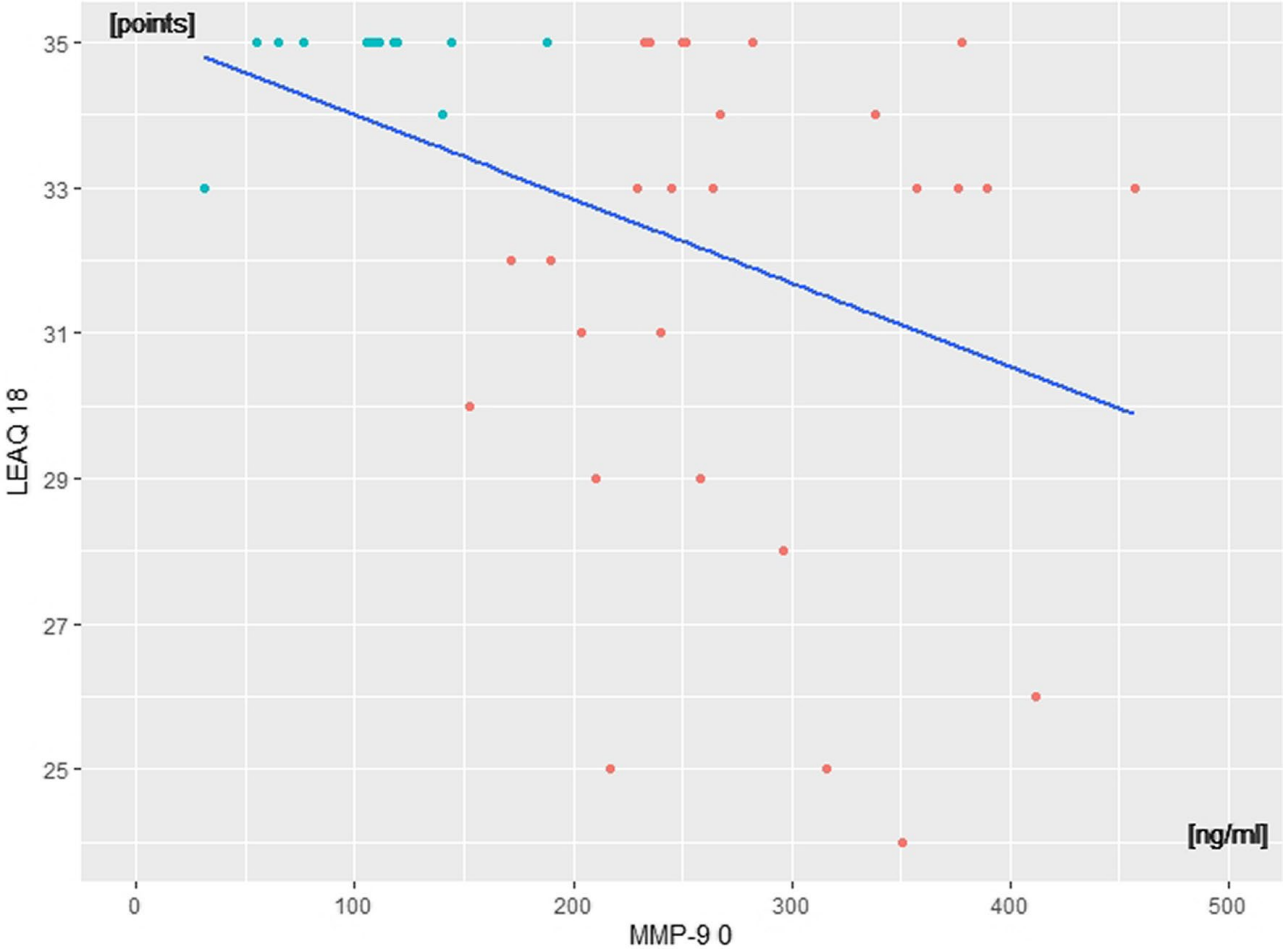
Distribution of MMP-9 plasma levels measured at cochlear implantation (MMP-9_0) and LEAQ scores at 18 months after CI activation (LEAQ_18) in the study group. Cluster 1—cyan dots; cluster 2—orange dots

Examining the data obtained in the correlation between MMP-9_0 and LEAQ_18, we extracted those patients who had higher and more homogeneous LEAQ_18 scores. The use of the PAM algorithm allowed us to identify two clusters: *n* = 13 in cluster 1 and *n* = 27 in cluster 2 (Fig. 1).

Mean age at CI activation in cluster 1 was 13.0 months (min = 7.8, max = 21.2, SD = 4.6) and in cluster 2 it was 13.9 months (min = 6.9, max = 21.8, SD = 4.1). In cluster 1, the younger activated formed 53.8% (7/13) of the group, while the older activated made up 46.2% (6/13) of the group. In cluster 2, the first made up 40.7% (11/27) of the group, and the latter 59.3% (16/27).

Based on these results, we propose that a MMP-9_0 plasma level threshold of 150 ng/ml separates the implanted children into two subgroups of good vs. poor performers.

Our protocol called for longitudinal observations of the mean values of LEAQ scores and plasma levels of MMP-9 to be made*.* Looking at each cluster separately, we see significant differences between them in terms of MMP-9_0 and MMP-9_8 scores and LEAQ_8 and LEAQ_18 scores (Table 3).

**Table 3:** Plasma levels of MMP-9 at all three follow-up intervals in cluster 1 (*n* = 13) and cluster 2 (*n* = 27). The *p* values show paired comparisons of mean values between the clusters

	LEAQ_0				LEAQ_8				LEAQ_18			
	Mean	Min	Max	SD	Mean	Min	Max	SD	Mean	Min	Max	SD
Cluster 1	6.7	0	19	6.6	30	22	33	3.0	34.8	33	35	0.5
Cluster 2	4.8	0	28	6.7	25.8	9	35	6.3	31.5	24	35	3.4
*p*-value	0.31				**0.03**				**< 0.01**			

	MMP-9_0 [ng/ml]				MMP-9_8 [ng/ml]				MMP-9_18 [ng/ml]			
	Mean	Min	Max	SD	Mean	Min	Max	SD	Mean	Min	Max	SD
Cluster 1	105.4	31.4	188.2	41.4	74.1	10.32	194.0	54.4	85.1	18.99	244.0	67.5
Cluster 2	280	152.3	456.7	78.1	167.8	26.1	490.4	136.6	139	138.9	512.3	114.3
*p*-value	**< 0.01**				**0.01**				0.11			

*min* minimum value, *max* maximum value, *SD* standard deviation

## Discussion

For the first time, the current study has carried out a longitudinal, prospective assessment of the plasma levels of MMP-9 in a homogenous cohort of congenitally deaf implanted children, and specifically excluding a number of factors that might potentially affect cochlear implantation outcomes.

We have shown that an MMP-9 plasma level of 150 ng/ml, measured at cochlear implantation, appears to be a reasonably effective threshold for differentiating good and poor performers later in their auditory development. We also find that the predictive value of MMP-9, measured at implantation, on the LEAQ score at 18 months also holds for the plasma level of MMP-9 measured at 8 months. However, LEAQ scores measured at cochlear implantation and at later intervals did not correlate with corresponding measurements of MMP-9.

### Grouping of good and poor performers

Based on MMP-9 levels measured at cochlear implantation, we have isolated two clusters of performers. Cluster 1 were those children who had an MMP-9 plasma level of below 150 ng/ml just before they started speech and language rehabilitation with their CI, and after 18 months they were among the best performers. Cluster 2 was those children who had higher levels of MMP-9 at cochlear implantation, and here one can see a considerable variance in their LEAQ scores at the end-point of observation. Since in both clusters, the mean values of age at CI activation carry a variance, one cannot attribute a direct effect of age on the variance of LEAQ score at 18 months. These findings lead us to conclude that an MMP-9 plasma level below 150 ng/ml at the time of cochlear implantation predisposes a child who has been diagnosed with DFNB1-related deafness to be a good performer, and is highly likely to reach full auditory development 18 months later. The same pattern exists for MMP-9_8 and LEAQ_18.

Nevertheless, there was one child in cluster 1 who started with an MMP-9 serum level of 188 ng/ml and who went on to score the maximum number of points in LEAQ 18 months later. Detailed analysis of this case revealed that the child was raised by very committed parents and was introduced into a rich, regular rehabilitation program. On LEAQ, the child had already scored 33 points at the 8-month follow-up, which suggests that the parents’ attitude played a big part in the treatment outcome.

### Temporal dynamics of MMP-9 in plasma

An advantage of a longitudinal study is that it allows one to analyze the impact of certain features, and how they interact over time. In our material, prospective observation of protein plasma levels in both clusters enabled us to see that MMP-9 concentrations decreased over time, although there were no significant differences between them. The natural, individual changes in concentrations of these proteins in plasma and brain tissue of human neonates and toddlers remain unknown, either under healthy or pathological conditions, and for this reason, we must rely on data from animals and human adults*.* However, Bjelosevic et al. report in their large study of levels of plasma proteins in healthy individuals of different ages that neonatal levels are very much different from those seen in 1–5 year olds or in adults [38]. Results of animal studies indicate that the protein is highly abundant, especially in the juvenile brain under dynamic development [11, 14]. In rats, MMP-9 concentrations in the brain, particularly in the hippocampus, are highest in the earliest postnatal period and decrease into adulthood [39, 40]. Kudo et al. reported on MM-9 protein plasma levels in human adults, and their results from healthy volunteers are much lower than in our subjects, showing mean values of 26.4 ng/ml versus 121.4 ng/ml here [25].

### Brain–blood plasma relation

There are several questions arising from our results. First, the peripheral levels of MMP-9 might not relate to the protein’s level in brain neuropil at all. However, some evidence emerging from studies of mice shows that MMP-9 cleaves collagen IV—a major component of the basal membrane of the cerebral vascular endothelium—and may thus change the permeability of the brain–blood barrier (BBB) [41]. It has been shown that levels of MMP-9 in animal brain cortex, cerebrospinal fluid, and serum are simultaneously coupled [39, 40, 42, 43]. In this context, despite existing evidence that the plasma levels of the protein can be influenced by its secretion from endothelium, we assume similar dynamics between brain and blood in humans [41, 43]. For now, no research tool is available to assess MMP-9 protein concentrations in the cortex of the human infant brain.

We suggest, although with extreme caution, that the decrease of MMP-9 levels observed in our clusters might be interpreted in analogy with data on Fragile X syndrome (FXS), a condition associated with cognitive deficits, anxiety, mood imbalance, and increased MMP-9 plasma levels [27]. A clinical trial of minocycline, which lowers MMP-9 levels, has shown that the drug can attenuate clinical features of FXS [28]. It is, therefore, possible to conclude that large decreases in peripheral MMP-9 levels, either pharmacologically induced or naturally occurring, might correspond with better neurocognitive conditions.

### Variance of MMP-9 plasma levels

Another question that needs to be addressed here is the observed variance across our cohort of MMP-9 plasma levels measured at cochlear implantation. It is known that MMP-9 activity remains under tight and complex regulation, both temporally and spatially, largely through gene expression, mRNA, and protein translation [14]. MMP-9 is a pleiotropic enzyme involved in numerous processes with a long list of targets, including growth factors, cell surface receptors, and cell adhesion molecules, which we think may explain the variance [11, 12, 43]. The biological mechanism behind MMP-9 responses to cochlear implantation remains unclear. It should be noted, however, that experimental studies have shown that both increased and decreased MMP-9 activity produces alterations in synaptic plasticity, learning, and memory [44, 45]. In fact, Wiera et al. directly demonstrated deficits in LTP (an electrophysiological model of synaptic plasticity) in gene knockout mice lacking MMP-9 activity, as well as in rats overexpressing the MMP-9 gene in neurons [46]. Furthermore, clinical studies of MMP-9 in schizophrenia (plasma levels as well as gene polymorphisms) have shown that both higher and lower levels of the enzyme may be associated with the disease [12].

### Limitations

It is important to underline that no data currently exist on the relation between MMP-9 concentration in brain and blood. However, our aim was not to elucidate the exact mechanisms of MMP-9 involvement in synaptic plasticity, instead, we were looking for a potential biomarker of neuroplasticity. At present, there are no objective tests for assessing auditory development in children up to 2 years old. We, therefore, need to accept the parents’ subjective rating as incorporated into the LEAQ score, since all current tests for young children rely on parental questionnaires. We have not taken into consideration the impact of family support on the likelihood of successful rehabilitation [2]. Detailed observation of rehabilitation would take much time and would require more frequent follow-ups. Ethnically, our cohort comprised only a Caucasian (Polish) population, so more diverse studies would be of great value in confirming the broader character of our results.

## Conclusions

We conclude that a plasma level of MMP-9 below 150 ng/ml at cochlear implantation indicates that an implanted and otherwise healthy child has a high chance of becoming a good auditory performer. However, that is not to say that CI children who have a higher MMP-9 serum level at implantation could not score well too, although the chances are lower, and obviously, these children will need additional, individual attention and effort during speech and language rehabilitation. In summary, we propose that MMP-9 is a biomarker for good auditory performance. Nevertheless, the regulation of MMP-9 is complex and the exact mechanisms by which MMP-9 affects the processes of memory and language formation following deafness treatment remain unknown. Further investigation of the cluster of children who started with plasma levels of MMP-9 above 150 ng/ml might help to discern additional factors, for example molecular partners of MMP-9, which are involved in the process of auditory neuroplasticity. In addition, longitudinal research on MMP-9 in bigger cohorts and with more reliable speech tests, extending perhaps to 5–6 years after CI activation, would add to our understanding of the role of MMP-9 in the molecular machinery of neuroplasticity.

## Data Availability

The datasets analyzed during this study are accessible on reasonable request from the corresponding author.
